# Erie County Medical Society

**Published:** 1863-02

**Authors:** Leon F. Harvey

**Affiliations:** Secretary


### ERIE COUNTY MEDICAL SOCIETY.

This Society met Tuesday, January 13, 1863, Dr. J. B. Samo, President, in the Chair. Dr. J. R. Lothrop was appointed Orator for the next meeting, and Dr. Parker of Clarence, as substitute. The annual election resulted as follows:

Dr. Charles Winne, President; Dr. C. C. Wyckoff, Vice President; Dr. Leon F. Harvey, Secretary; Dr. Wm. King, Treasurer; Dr. J. B. Samo, Librarian.

*Primary Board.*—Dr. C. L. Dayton, Dr. George Abbott, Dr. P. P. E. Tobie.

*Censors.*—Dr. J. Boardman, Anatomy, Physiology and Surgery; Dr. J. Cronyn, Practice of Medicine and Obstetrics; Dr. O. K. Parker, Materia Medica and Botany; Dr. J. R. Lothrop, Chemistry and Pharmacy; Dr. H. M. Congar, Medical Jurisprudence and Pathology.

No business of importance was transacted,

LEON F. HARVEY, Secretary.

